# Specially Communicated by an M.O.H.

**Published:** 1920-06-12

**Authors:** 


### LORD DAWSON'S SCHEME IN PRACTICE.

### Specially Communicated by an M.O.H.

SOMEONE recently formulated a plan to improve the transport system of this country, and so impressed the authorities by its possibilities that they decided to hold a public inquiry on the scheme. It was an ideal one and no doubt its adoption would have completely reformed our transport, but it had one great fault—namely, that it was too sweeping and meant scrapping the present system and the expenditure of untold sums in acquiring lands, building railways, sheds, wharves, and machinery, not to speak of the chaos likely to ensue during the transition period. *The Times* summed up the system as being too elaborate and falling by its own weight.

We all have our ideals and the world would be poor without them, but those of us who have spent a large part of our lives in the public service have come to look with some distrust on many of the schemes which have recently emanated from high places, since they all suffer from the fault of the transport system mentioned and have already fallen or are likely to fall through their own weight. These remarks apply to the report on the medical services now before me. Lord Dawson's scheme may be ideal in many respects, but it is far too sweeping, elaborate, and costly, and, like the transport scheme, may bring about its own downfall.

### THE PRESENT SYSTEM'S FATE.

In the first place it means scrapping our present local sanitary authorities, our present Public Health services built up laboriously during the last eighty or ninety years, our present hospital service, the insurance system with its panel of doctors, a large portion of our medical educational system, and the building of new and costly health centres, primary and secondary, new hospitals—a complete reorganisation of our voluntary system and the glorifying of specialists of all sorts. In other words, it savours too much of an elaborate State system, and medical men as well as patients will find themselves in the grip of a complicated and far-seeking organisation very much like the R.A.M.C. Such an organisation may be very good for an army—but will not suit the civilian, for it suffers from over-organisation. It is true there are two concessions to freedom—*viz.*, the retention of voluntary hospitals and "freedom of choice" of doctor by the patient; but I suspect that as this system is elaborated even these will disappear, and we will stand before a complete State medical service which will sweep the whole population into its net.

### OTHER POSSIBILITIES.

I have searched in vain for some attempt to improve our present medical and health system, or to adapt these new proposals to it. I am far from thinking that our present system is perfect. Clinical laboratories and specialists should be available outside our large hospitals and teaching schools for the middle and lower classes, but these could be fitted on to our present system at very little trouble and expense. Under the panel system people now have an actual choice of doctor, but doctors could and should be limited as to the number they have on their list. The prevention and treatment of tuberculosis could be greatly improved by utilising and improving our present dispensaries and the provision of more sanatoria. There are many voluntary organisations, especially in maternity and child-welfare, which are being gradually co-ordinated with municipal work or else being absorbed by the local sanitary authority. School clinics are being undertaken by county councils, examination of school children, etc., and all this is being done without disturbing the communities they benefit.

### THE INDIVIDUAL DOCTOR.

One good point made by the Council is in paragraph 96, where they state that the medical profession should come into "organic relation with the health administration." This is excellent—but he should come in not as a subordinate or kind of "orderly" in a big machine, but as a voluntary worker with a real voice in directing that machine. It may be replied that the Council have provided for this, inasmuch as he is to be co-opted on the health authority, but the scheme suggested is of such a nature that when once established it can be transferred bodily to the work and develop into an elaborate State system controlled by a central authority—a kind of civil G.H.Q.

Such a State system as here adumbrated should certainly not be received with open arms by the population of these islands in their present frame of mind. Their experience of State control with its costly blunders is too recent to make them desire to have more. The "team-work" done in the Army has certainly shown the value of co-operation. Let us by all means co-operate and organise, but do not let us fall into the greatest error of over-organisation. One of our late enemies owes his downfall—in part at least—to over-organisation, and let us avoid this pitfall, contenting ourselves with evolution, rather than revolution, in our medical services.

